# Enhanced STEAP4 Ubiquitination in Obesity: Insights From Combined Proteome and Ubiquitylome Analysis of Visceral Adipose Tissue

**DOI:** 10.1016/j.mcpro.2025.101474

**Published:** 2025-11-27

**Authors:** Yuhao Li, Jie Li, Genyao Wang, Shushu Yang, Dong Liu, Hao Wen, Mengjun Zhang, Chengjie Duan, Meiling Yu, Shufeng Wang, Sheng Guo, Xiaoling Chen, Li Wang

**Affiliations:** 1 Institute of Immunology PLA, Army Medical University (Third Military Medical University), Chongqing, China; 2 Department of Pharmaceutical Analysis, College of Pharmacy, Army Medical University (Third Military Medical University), Chongqing, China

**Keywords:** obesity, proteome, STEAP4, ubiquitylome, VAT

## Abstract

Obesity remains a worldwide health issue, with visceral adipose tissue as a leading driver of this pathology. As the executors of biological functions in living cells, proteins have their activity regulated by diverse post-translational modifications, including ubiquitination. However, obesity-related changes in ubiquitination of visceral adipose tissue (VAT) proteins are still poorly understood. Here, we obtained the global proteomic and ubiquitylomic data of epididymal VAT from lean and obese male mice by mass spectrometry. Our proteomic analyses revealed significant changes of metabolic pathways involved in fatty acid, acyl-CoA and branched chain amino acids metabolism in obese VAT. Intriguingly, a comparative analysis of proteomic and ubiquitylomic data highlighted discordance in the quantity changes of certain proteins and their ubiquitination levels. Notably, STEAP4 exhibited a markedly reduced protein level coupled with an enhanced K48-linked ubiquitination, suggesting a potential role for ubiquitination-mediated proteasome degradation in VAT dysfunction. Further *in vitro* experiments revealed that knockdown of STEAP4 in adipocytes impaired mitochondrial function of 3T3-L1 adipocytes. Collectively, this study introduces the first combined proteomic and ubiquitylomic examination of murine VAT, offering novel insights and potential therapeutic targets for obesity.

## Highlights

• Combined proteome and ubiquitylome analysis of visceral adipose tissue during obesity.
• STEAP4 is degraded via ubiquitin-proteasome in visceral adipose tissue during obesity.
• STEAP4 knockdown impairs adipocyte mitochondrial function, indicating therapeutic value.

## Graphical Abstract

**Figure:**
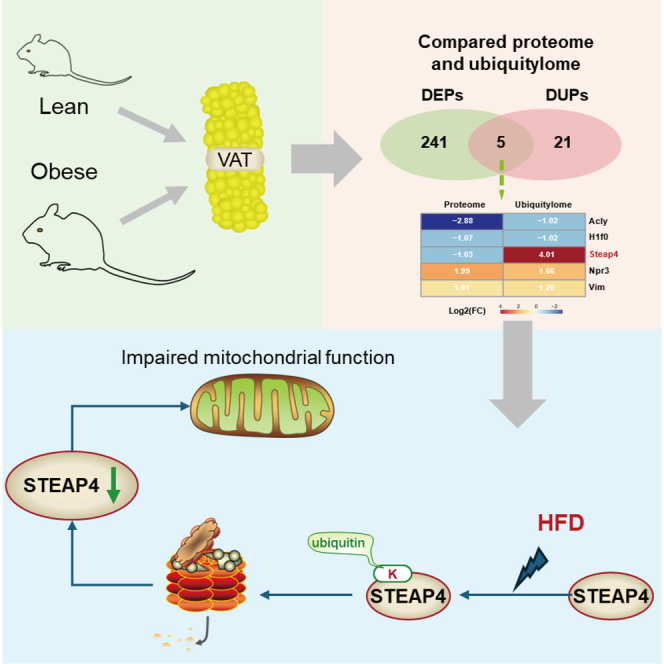

## In Brief

This study presents the combined proteomic and ubiquitylomic analysis of visceral adipose tissue in lean and obese mice, identifies ubiquitination-mediated STEAP4 downregulation in obesity, and links STEAP4 to adipocyte mitochondrial function, offering obesity therapeutic insights.

Obesity, characterized by abnormal or excessive fat accumulation, poses a significant risk for metabolic syndrome including insulin resistance (IR), type 2 diabetes mellitus (T2DM), fatty liver, hypertension and cardiovascular disease, as well as non-metabolic disorders such as cancer and infectious diseases (1, 2). In 2022, 43% adults aged 18 and older were overweight, and 19% were classified as obesity (3). The soaring prevalence of obesity is exacerbating the global disease burden.

Adipose tissue (AT) plays a crucial role in regulating systemic energy levels. The risk of obesity is more closely linked to the distribution of body AT rather than its total amount (4). AT is divided into subcutaneous adipose tissue (SCAT) and visceral adipose tissue (VAT), which differ in biochemical and metabolic properties. SCAT serves as a natural energy reservoir, functioning as a physiological buffer for excess energy intake with limited energy expenditure (4). When the storage capacity or proliferation of SCAT is impaired due to genetic and environmental stress, AT begins to accumulate in the visceral areas to form VAT, which leads to the development of metabolic syndrome (5). However, the metabolic pathways and biological processes disrupted by nutrient overload within VAT are not entirely clear.

In recent years, proteomics is widely used for finding potential biomarkers and targets for diseases. So far, numerous proteomics studies on AT in humans and animals have been conducted, revealing insights into the molecular basis of obesity (6, 7). After transcription and translation, proteins often undergo post-translational modifications (PTMs) to perform their crucial function. So integrated analysis of proteomics and PTMs omics can provide guidance to better understand the mechanism of the disease. For instance, an integrated analysis of organellar proteome and phosphor-proteome of liver from obese and lean mice provides a crucial and global understanding of liver dysfunction caused by nutrient overload (8). Ubiquitination is a vital PTMs process which plays an important role in protein degradation, endocytosis, signal transduction and so on (9). Given that there was no ubiquitylomics data of VAT, we employed liquid chromatography-tandem mass spectrometry (LC-MS/MS)-based proteome and ubiquitylome to detect changes in protein and ubiquitination events in VAT of high fat diet (HFD)-induced obese (DIO) mice and control lean mice. In brief, this study provides a combined analysis of VAT proteome and ubiquitylome remodeling in HFD-induced obesity and discovers that HFD upregulates the ubiquitination level of six-transmembrane epithelial antigen of prostate 4 (STEAP4) in the VAT of DIO mice, leads to the degradation of STEAP4 *via* the ubiquitin-mediated proteasomal pathway, thereby causing a decrease in the protein level of STEAP4, which further results in reduced mitochondrial function in adipocytes.

## Experimental Procedures

### Experimental Design and Statistical Rationale

**Fig. 1:**
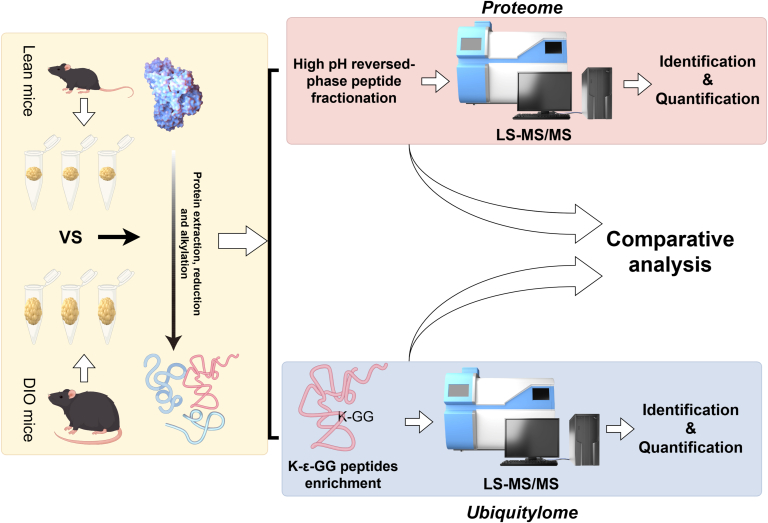
**Overview of the workflow for combined proteome and ubiquitylome profiling.** The experimental workflow for the proteome and ubiquitylome analysis of proteins extracted from epididymal white adipose tissue of C57BL/6 mice fed with HFD or NCD for 8 weeks, starting at 4 weeks of age. DIO, diet-induced obese; HFD, high-fat diet; NCD, normal chow diet.

The aim of this study was to explore the alterations in the overall proteome and ubiquitylome of VAT during the process of obesity, as well as to identify potential target proteins related to this process. The workflow was presented in Figure 1. VAT were isolated from both obese and normal C57BL/6J mice. Subsequently, proteins were extracted from these tissues and subjected to trypsin digestion, peptide enrichment, and mass spectrometry analysis. Each sample group included three biological replicates. For the proteomic analysis, high pH reversed-phase peptide fractionation method was employed to enrich peptides. Regarding the ubiquitin proteomics, due to the low abundance of ubiquitin-modified peptides in the total proteome, a di-Gly remnant (K-ϵ-GG) specific antibody was utilized to enrich the ubiquitin-modified peptides (10).

The false discovery rate (FDR) cutoff values for proteins, peptides, and ubiquitination sites were set at 0.01. Uncorrected *p* value of <0.05 and a fold change (FC) of ≥2 were considered significant. Fisher's exact test was employed for functional enrichment analysis. Independent Student's t-tests were used for comparisons between the two groups. *p* value of <0.05 was used as the critical value for both statistical analyses. All statistical analysis was performed using GraphPad Prism 8 Software (https://www.graphpad.com/scientific-software/prism/). Two-way ANOVA was used for multiple comparisons followed by the Sidak multiple comparisons tests. All data are presented as the mean ± SD. *p* < 0.05 (∗), *p* < 0.01 (∗∗), *p* < 0.001(∗∗∗) and *p* < 0.0001(∗∗∗∗) were considered statistically significant.

### Animals

Male WT C57BL/6J mice (4-week-old) were purchased from Beijing HFK Bioscience (Beijing, China) and were housed in specific pathogen-free facilities with a 12-h light-dark cycle. Sterilized water and a normal chow diet (NCD, D12450 B 10% Kcal from fat, Research Diets) or a high-fat diet (HFD, D12492, 60% Kcal from fat, Research Diets) were allowed free access for mice for 8 weeks. Mice were fasted of food for 4 h in the morning followed by the anesthetization before necropsy. All procedures were approved by the Institute Animal Care and Use Committee of Army Medical University (no. AMUWEC20218024).

### Sample Preparation for Proteome

Mice were subjected to cardiac exsanguination followed by perfusion of the heart with a 0.9% NaCl solution. Subsequently, the epididymal white adipose tissue (epiWAT) was harvested. SDT lysate (4% (w/v) SDS, 100 mM Tris/HCI pH7.6, 0.1 M DTT) was added to 60 mg epiWAT sample (with each sample being a pool of tissue from three individual mice) for protein extraction followed by BCA assay for protein quantification. An appropriate quantity of protein sample was digested with trypsin using Filter aided proteome preparation (FASP) method to obtain enzymatically hydrolyzed peptides (11). The peptide was desalted using C18 Cartridge and lyophilized. The proteomic peptides were re-solubilized with 40 μl of 0.1% formic acid solution and quantified (OD280).

### Sample Preparation for Ubiquitylome

To enrich ubiquitinated peptides, proteins were extracted from 200 mg epiWAT (with each sample being a pool of tissue from three individual mice) using the Urea/Tris-HCl (8 M Urea, 100 mM Tris/HCl, pH 8.5) lysis method, and then the Bradford method was used for protein quantification. For each sample, 20 μg of protein was taken and mixed with 5 × loading buffer respectively, followed by boiling in water bath for 5 min, and then subjected to 12.5% SDS-PAGE electrophoresis (constant current of 14 mA for 90 min) and Coomassie brilliant blue staining. For all samples, DTT was added to a final concentration of 10 mM, and the samples were placed in a constant-temperature mixer (600 rpm, 37 °C) for 1.5 h, then taken out and cooled to room temperature. IAA was added to a final concentration of 50 mM, and the reaction was carried out in the dark for 30 min 4-fold volume of 50 mM Tris-HCl (pH 8.0) was added to dilute the UA concentration to 2 M. Trypsin was added at a protein: Trypsin mass ratio of 50:1, and enzymatic digestion was performed at 37 °C overnight (15–18 h). Trifluoroacetic acid (TFA) was added to a final concentration of 0.1%, and the volume of 10% TFA was adjusted to make the sample pH ≤ 3. The peptides were desalted using a C18 SPE Cartridge and then lyophilized.

For the affinity enrichment of ubiquitinated peptides, 1.5 mg lyophilized peptides dissolved in pre-cooled immunoaffinity purification (IAP) buffer were co-incubated with preconditioned Anti-K-ε-GG antibody-bead complexes (PTMScan Ubiquitin Remnant Motif (K-ε-GG) Kit, Cell Signaling Technology) for 1.5 h at 4 °C. The antibody is pre-crosslinked to the beads by the manufacturer (per kit protocol), requiring no additional crosslinking. Supernatants were discarded by centrifugation for 30 s at 2000*g*. The beads were washed three times with pre-cooled IAP buffer and three times with pre-cooled ddH2O. After cleaning, Anti-K-ε-GG antibody beads were added with 40 μl 0.15% TFA twice at room temperature for 10 min. Centrifuge at 2000*g* for 30 s, recover supernatant and desalinate by C18 STAGE Tips.

### LC-MS/MS Analysis

#### Sample Preparation and High-pH RP Fractionation

The lyophilized sample (100 μg) was reconstituted in 300 μl of 0.1% TFA and fractionated using high-pH reverse-phase chromatography. Briefly, a spin column was preconditioned by centrifugation at 5000*g* for 2 min to remove storage solution, followed by two washes with 300 μl of acetonitrile (ACN) and two washes with 300 μl of 0.1% TFA (5000*g*, 2 min each). The sample was loaded onto the column and centrifuged at 3000 g for 2 min; the flow-through (F0) was collected. The column was washed with 300 μl of water (3000*g*, 2 min), and the wash fraction was discarded. Sequential elution was performed using 300 μl of increasing ACN concentrations in 0.1% triethylamine (TEA): 5% (F1), 7% (F2), 9% (F3), 11% (F4), and 50% (F5), each centrifuged at 3000*g* for 2 min. Fractions were vacuum-dried overnight at 25 °C and reconstituted in 20 μl of 0.1% formic acid for LC-MS/MS analysis. For proteomic analysis, 2 μl of each reconstituted fraction was injected into the LC-MS/MS system, while 6 μl was used for ubiquitomic analysis. The proteins were detected by Label Free Quantitative proteomics technology (Shanghai Applied Protein Technology).

#### Chromatographic Gradient

The separation was performed for all samples using the HPLC liquid phase system Easy nLC with nanoliter flow rate, with identical mobile phase constituents for both proteomic and ubiquitomic analyses: Buffer A consisted of a 0.1% formic acid-water solution, while Buffer B was a 0.1% formic acid-acetonitrile solution (84% acetonitrile). The chromatographic column was initially equilibrated with 95% of Buffer A. Samples were loaded onto the pre-column (Thermo Fisher Scientific Acclaim PepMap 100, 10 μm × 2 cm, nanoViper C18) *via* an autosampler and subsequently separated on the analytical column (Thermo Fisher Scientific EASY column, 10 cm, ID 75 μm, 3 μm, C18-A2) at a flow rate of 300 nl/min. Distinct gradient elution strategies were implemented based on the analysis type: for proteomic profiling, a 1H gradient was employed, with the gradient program comprising linear increases from 5% to 8% Buffer B (0–3 min), 8% to 26% Buffer B (3–43 min), 26% to 40% Buffer B (43–48 min), and 40% to 90% Buffer B (48–50 min), followed by isocratic elution at 90% Buffer B (50–60 min); for ubiquitomic analysis, a 2H gradient was utilized, with an initial linear increase from 2% to 8% Buffer B (0–3 min), followed by a prolonged gradient from 8% to 40% Buffer B (3–110 min), a rapid increase to 90% Buffer B (110–115 min), and isocratic maintenance at 90% Buffer B (115–125 min).

#### Mass Spectrometry Parameters

The mass spectrometer was operated in data-dependent acquisition (DDA) mode. Under the positive ion detection mode, the precursor ion scanning range was set to 300 to 1800 m/z. The resolution of the full-scan (MS1) was 70,000 at 200 m/z. The automatic gain control target was set to 1,000,000 and the maximum ion injection time (Maximum IT) was 50 ms. The dynamic exclusion time was set to 60 s. The mass spectrometry acquisition strategy was that 20 MS2 scans were acquired after each full scan. The MS2 activation type was high-energy collisional dissociation, with an isolation window of 2 m/z. The resolution of the MS2 was 17,500 at 200 m/z, and the normalized collision energy was set to 30 eV. The underfill ratio was 0.1%, so as to realize the effective acquisition and analysis of the mass-to-charge ratios of peptides and peptide fragments.

### Database Search

We used data-dependent acquisition mode for label-free quantification. A Q-Exactive mass spectrometer was used for the LC-MS/MS analysis. The original data were RAW files, and the MaxQuant software (version 1.5.3.17; https://www.maxquant.org/) was used for database identification and label-free quantitative analysis.

Trypsin was designated as the cleavage enzyme for proteomic peptides, with a maximum of two allowed missed cleavages. Mass error was set to 6 ppm for main search, 20 ppm for first search and MS/MS tolerance. Fixed modification was specified for carbamidomethylation on Cys, while variable modifications were specified for oxidation on Met. Protein and peptide were assigned FDR thresholds of 1%. Swiss_mouse_17016_20190314.fasta (containing 17,016 entry proteins) was used as database.

Up to two missing cleavages were allowed for Trypsin as the specified cleavage enzyme for peptides containing ubiquitinated Lys sites. Mass error was set to 6 ppm for main search, 20 ppm for first search and MS/MS tolerance. Fixed modification was assigned to Cys with carbamidomethylation, while variable modifications were assigned to Met with oxidation, Lysine with GlyGly, and Met with oxidation. The specified thresholds for protein, peptide, and modification site in FDR were set at 1%. Uniprot_Mus_musculus_87890_20200217.fasta (containing 87,890 entry proteins) was used as database.

The intensities of the peptides were subsequently analyzed using ion chromatograms *via* Xcalibur software (Thermo Fisher Scientific). For proteins identified based on a single unique peptide, their annotations are supported by the corresponding spectra, which have been deposited in the PRIDE partner repository under the dataset identifier PXD060435.

### Bioinformatic Analysis

To evaluate statistical significance, stringent methodologies were employed to guarantee the dependability of the findings. For data filtering, only protein groups and ubiquitinated peptides detected in ≥50% of replicates within each experimental group (*i.e.*, ≥2 out of three samples in either the NCD or HFD group) were included in quantitative analysis. For imputation of missing values, missing data points were imputed using the K-Nearest Neighbor method in both proteomic and ubiquitinomic analyses.

For differential protein analysis in the proteome and differential ubiquitination modification analysis in the ubiquitinome, we defined differentially expressed proteins (DEPs) or differentially ubiquitinated proteins (DUPs) as those exhibiting a fold change ≥2 and an uncorrected *p*-value <0.05. In enrichment analyses, the complete species proteome served as the background protein set; pathways with an uncorrected *p*-value <0.05 were considered significantly enriched. GO enrichment analysis and KEGG pathway enrichment analysis for DEPs were performed as follows: Functional annotation was conducted using Blast2GO (for GO) and KAAS (for KEGG), respectively, followed by Fisher’s exact test for significant enrichment. KEGG pathway enrichment, WikiPathway enrichment and Mammalian Phenotype Ontolog enrichment analyses were performed using the STRING database (v11.5). Regarding network analysis, the topological framework of protein-protein interaction networks was constructed using STRING (v11.5) and subsequently visualized and analyzed within Cytoscape (v3.9.1).

### Hematoxylin and Eosin Stain

ATs were collected and fixed in fatty tissue fixative solution for 24 h. Following paraffin embedding and sectioning, tissues were stained with hemotoxylin and eosin. Adipocyte size was measured using lmageJ software (https://imagej.net/ij/).

### Insulin and Glucose Tolerance Testing

Glucose tolerance test (GTT): mice were fasted for 16 h and then injected with glucose (2 g/kg) intraperitoneally. Blood glucose was measured at fixed times from the tail vein using a blood glucose meter (ONETOUCH Ultra).

Insulin tolerance test: mice fasted for 4 h were intraperitoneally injected with insulin (Novolin; 0.75 U/kg), and blood glucose concentrations were measured at designated time points.

### Western Blotting

Western blotting was performed as previously described (12). Briefly, epiWAT from mice fed with HFD or NCD (n = 3 per group) was lysed in RIPA buffer (50 mM Tris-HCl pH7.4, 1% Nonidet P-40, 0.25% Na-deoxycholate, 150 mM NaCl, 1 mM EDTA) containing complete protease inhibitor mixture (Roche Life Sciences) on ice, followed by centrifugation and denaturation. The proteins were separated by electrophoresis and transferred to PVDF membranes. Protein expression was normalized using the total protein normalization kit No-Stain Protein Labeling Reagent (A44449, Thermo Fisher Scientific). Then, the membranes were incubated with primary antibodies at 4 °C overnight and secondary antibodies at room temperature for 1 h before being blocked in 5% skimmed milk for 1 h at room temperature. After that, the chemiluminescence reagent (Amersham, Freiburg, Germany) was employed to detect the protein levels. The primary antibodies were used: STEAP4 (FNab08319, FineTest), GAPDH (GB15004, Servicebio). The following secondary antibodies were used: HRP-labeled Goat Anti-Rabbit IgG (H + L) (A0208, Beyotime). Three biological replicates of each group were detected.

### Immunoprecipitation

To identify ubiquitination, we conducted immunoprecipitation following established protocols (12). Pooled epiWAT samples (0.2 mg) from several obese or lean mice were washed twice with ice-cold phosphate-buffered saline and lysed in 1.2 ml immunoprecipitation (IP) lysis/wash buffer (2 × 50 ml, pH7.4, 25 mM Tris, 150 mM NaCl, 1 mM EDTA, 1% NP40, 5% glycerol) in the presence of 10 μM MG132 (a proteasome inhibitor). The lysates were centrifuged at 12,000*g* for 10 min at 4 °C and the supernatant was precleared with 20 μl Pierce Protein A/G Magnetic Beads (Thermo Fisher Scientific) for 2 h at 4 °C to remove non-specific binding. 5 μg of STEAP4-specific goat polyclonal antibody (NB100–68162, Novusbio) was incubated with 25 μl Pierce Protein A/G Magnetic Beads using the Pierce Crosslink Magnetic IP/Co-IP Kit (Thermo Fisher Scientific, #88805) for 2 h and followed by cross-linking with DSS for 30 min, and three washes with Elution buffer (pH=2.0) and pre-cooled IP lysis/wash buffer. The magnetic bead-Ab complex was mixed with the lysates and incubated at 4 °C overnight. The antigen STEAP4 was obtained by washing and elution with IP lysis/washing buffer and 50 μl elution buffer (pH2.0) and neutralizing pH with 5 μl Neutralization Buffer (pH8.5). Immunoprecipitates were boiled in 5×SDS sample buffer for 3 min, resolved by SDS-PAGE, and immunoblotted overnight using specific antibodies against STEAP4 (1:1000, FineTest, rabbit) and Lys48-specific Ubiquitin (clone EP8589, Abcam, 1:1000, rabbit) at 4 °C. The visualization of proteins was achieved by employing a goat anti-rabbit secondary antibody conjugated to HRP (A0208, Beyotime, diluted to 1:5000, incubated for 2 h at room temperature) and a chemiluminescence detection system.

### RNA Extraction and Quantitative Real-time PCR (qPCR)

Total RNA was extracted from target cells using the SteadyPure Quick RNA Extraction Kit (Accurate Biology, AG21023) following the manufacturer's protocol. cDNA synthesis was then performed with the Evo M-MLVRT Mix Kit (Accurate Biology, AG11728). Subsequent qPCR analysis utilized the Premix Pro Taq HS qPCR Kit (Accurate Biology, AG11701). Target gene mRNA expression levels were normalized against *Actin*. qPCR primers sequences are as follows: *Steap4*: forward 5′-GGGAAGTCACTGGGATTGAAAA-3′, reverse 5′-CCGAATAGCTCAGGACCTCTG-3′; *Actin*: forward 5′-GGCTGTATTCCCCTCCATCG-3′, reverse 5′-CCAGTTGGTAACAATGCCATGT-3′.

### 3T3-L1 Cell Culture and Differentiation

3T3-L1 cells were maintained in growth medium (DMEM containing 10% FBS and 1% penicillin-streptomycin) until confluence was achieved. Differentiation was initiated by replacing the medium with growth medium containing the adipogenic cocktail: 1 μM dexamethasone (Selleck, S1322), 0.5 mM isobutylmethylxanthine (MCE, HY-12318), 5 μg/ml human insulin (Beyotime, P3376), and 2 μM rosiglitazone (Selleck, S2556). After 2 days, the induction cocktail was removed and the cells were switched to growth medium containing 5 μg/ml human insulin for a further 2 days.

### siRNA Transfection

*Steap4* expression was knocked down using a pool of three distinct siRNAs targeting the *Steap4* gene (HanBio, sequences: siRNA1 forward 5′-GGUCCUGAGCUAUUCGGAATT-3′, reverse 5′-UUCCGAAUAGCUCAGGACCTT-3′; siRNA2 forward 5′-GAUGCUUUGCAGAAAGCAATT-3′, reverse 5′-UUGCUUUCUGCAAAGCAUCTT-3′; siRNA3 forward 5′-CGUCAGUAACAACCGCAAATT-3′, reverse 5′-UUUGCGGUUGUUACUGACGTT-3′) alongside a non-targeting control siRNA (siRNA-NC forward 5′-UUCUCCGAACGUGUCACGU-3′, reverse 5′-ACGUGACACGUUCGGAGAA-3′). Transfections were performed using RFect siRNA Transfection Reagent (Cat#: 11013, Baidai Biotechnology) according to the manufacturer's protocol. Briefly, 1 × 10^5^ cells per well (24-well plate) were transfected by incubating for 20 min at room temperature with a complex formed from 15 pmol siRNA, 5 μl RFect reagent, and 200 μl serum-free DMEM; this complex was then added to cells in 300 μl serum-free DMEM. After 6 h, the medium was replaced with complete medium containing 10% FBS. Knockdown efficiency was assessed by qPCR and Western blotting 72 h post-transfection, and cells were also used for downstream experiments at this timepoint.

### Flow Cytometric Analysis

Following trypsinization, cells were washed with 500 μl wash buffer (1 × PBS supplemented with 1% BSA) by centrifugation at 300*g* for 5 min. Cell pellets were resuspended in 100 μl wash buffer and stained with LIVE/DEAD Fixable Near-IR Dead Cell Stain Kit (L34972, Invitrogen) at a 1:200 dilution for 30 min at room temperature in the dark to discriminate viable cells. Subsequently, cells were incubated with MitoSOX Red Mitochondrial Superoxide Indicator (HY-D1055, MedChemExpress) at a 1:1250 dilution for 30 min at 37 °C to assess mitochondrial reactive oxygen species (ROS) production. Mitochondrial membrane potential was evaluated using tetramethylrhodamine ethyl ester (TMRE; HY-D0985A, MedChemExpress) at a 1:5000 dilution for 30 min at 37 °C in the dark. Flow cytometric data were acquired on a BD Canto II analyzer, BD LSRFortessa, or CytoFlex LX (Beckman Coulter) and analyzed using FlowJo software (version 10, BD Biosciences, https://www.flowjo.com/).

### ATP Measurement

ATP content was measured with ATP determination kits (Nanjing Jiancheng Bioengineering Institute, Nanjing, Jiangsu province, China). The final ATP content of each sample was normalized to its protein concentration with the BCA protein detection kit (Beyotime, China).

## Results

### Establishment of High Fat Diet-Induced Obesity (DIO) Mice Model

**Fig. 2:**
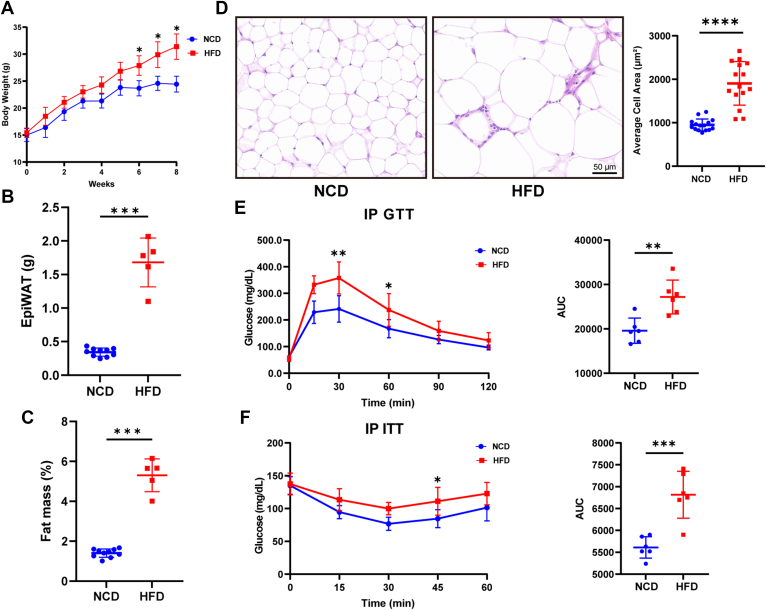
**Establishment of high fat diet-induced obese mice model.***A*, body weight changes of C57BL/6 mice fed with HFD or NCD for 8 weeks, starting at 4 weeks of age. Comparison of (*B*) epiWAT weight and (*C*) mass (the ratio of epiWAT to body weight) of 12-week-old C57BL/6 mice fed with HFD (n = 5) or NCD (n = 10). *D*, HE staining of epididymal white adipose tissue (400x). *E*, IPGTT and (*F*) IPITT results for 12-week-old C57BL/6 mice fed with HFD (n = 5) or NCD (n = 5). Area underneath curve of (*E*) IPGTT and (*F*) IPITT. Data represent the mean ± SD. ∗*p* < 0.05, ∗∗*p* < 0.01, ∗∗∗*p* < 0.001 and ∗∗∗∗*p* < 0.0001 between indicated group; Unpaired *t* test was used for *B*, *C*, *D*, and area underneath curve *in E* and *F;* Two-way ANOVA with Sidak multiple comparisons tests was used for *A*, *E* and *F* The results shown are representative of one of two independent experiments. HFD, high-fat diet; NCD, normal chow diet; epiWAT, epididymal white adipose tissue; IPGTT, intraperitoneal glucose tolerance test; IPITT, intraperitoneal insulin tolerance test. ITT, Insulin tolerance test.

After 8 weeks of HFD feeding, the DIO mice model was well established. Compared to NCD fed lean mice, DIO mice showed significantly increased body weight and the epididymal white adipose tissue (epiWAT, visceral adipose tissue samples) mass (Fig. 2, *A–C*). HE staining of VAT showed that DIO mice had larger lipid droplets and increased fat cell volumes compared to lean mice (Fig. 2*D*). The glucose tolerance test and insulin tolerance test results indicated that HFD impaired glucose tolerance (Fig. 2*E*) and insulin sensitivity (Fig. 2*F*), resulting in insulin resistance.

### HFD Induced Changes in the Proteome Profile of VAT

**Fig. 3:**
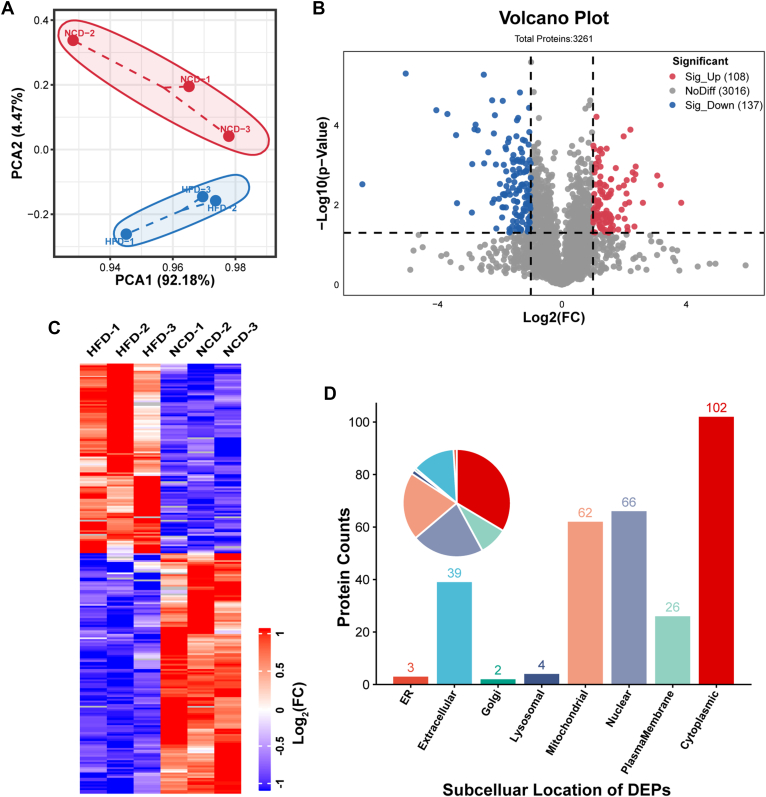
**Proteome profile changes of VAT from obese and lean mice.***A*, principal component analysis of all samples. *B*, volcano plot illustrates significantly up-regulated (*red dots*) and down-regulated (*blue dots*) proteins in VAT of mice between HFD and NCD groups, with log2 fold-change on the x-axis and *t* test significance [-log_10_(*p* value)] on the y-axis. *C*, clustering analysis of significantly differently expressed proteins in proteome. *D*, subcellular localization of significantly DEPs. VAT, visceral adipose tissue; HFD, high-fat diet; NCD, normal chow diet; DEPs, differentially expressed protein.

**Fig. 4:**
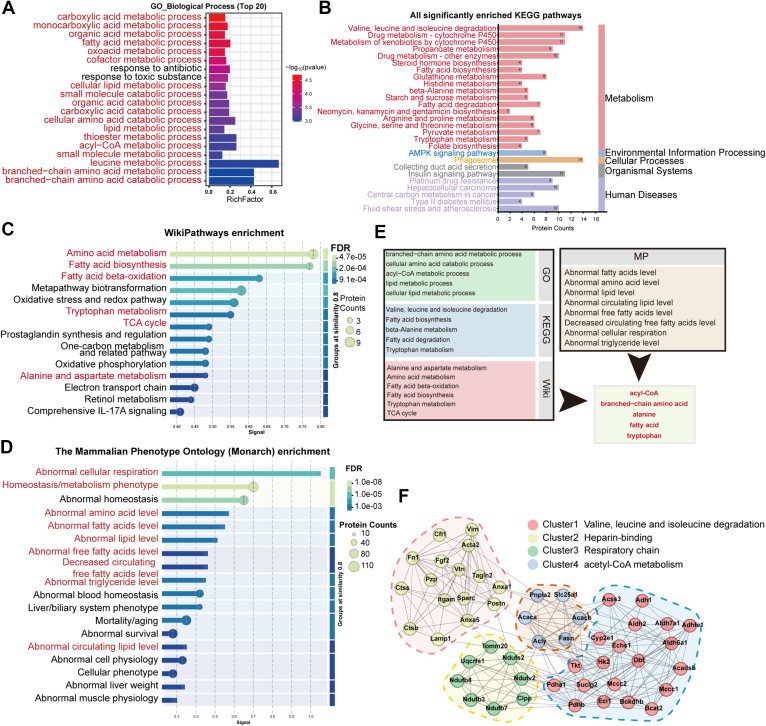
**Functional enrichment analysis of significantly differently expressed proteins in proteome.***A*, gene ontology biological process enrichment analysis of these DEPs. *B*, KEGG enrichment analysis of these DEPs. *C*, WikiPathways enrichment analysis of these DEPs. *D*, mammalian Phenotype Ontology enrichment analysis of these DEPs. *E*, overlap of pathways with similar GO, KEGG, WikiPathways, and MP terms. *F*, protein-protein interaction network of top 50 DEPs with the highest interaction scores. DEPs, significantly differentially expressed protein.

Our comprehensive proteome analysis identified a total of 3789 proteins and 28,681 peptides across all VAT samples from mice fed either NCD or HFD (Supplemental Table S1). Principal component analysis for each replicate in our proteome profile showed distinct separations between the VAT samples from both groups (Fig. 3*A*). Using a cutoff criterion with a FC in the protein abundance ≥2 and uncorrected *p* value < 0.05, we acquired 108 up-regulated proteins and 137 down-regulated proteins (Fig. 3*B*). Hierarchical cluster analysis of these 245 DEPs, clearly distinguished the two groups (Fig. 3*C*), thereby validating the reliability of our analysis. Subcellular localization showed that all DEPs were mainly localized in cytoplasmic, followed by nuclear and mitochondrial (Fig. 3*D*). Biological process terms enrichment analysis revealed that these DEPs were mainly enriched in biological processes related to metabolism (Fig. 4*A*). Kyoto Encyclopedia of Genes and Genomes (KEGG) pathway enrichment analysis also highlighted that 18 out of 28 significantly enriched pathways were related to substance metabolism (Fig. 4*B*). Consistently, WikiPathways enrichment analysis also showed substantial changes in metabolism pathways (Fig. 4*C*). Mammalian Phenotype Ontology (MP) enrichment analysis unveiled the presence of abnormal metabolites levels in VAT of DIO mice (Fig. 4*D*). Notably, acyl-CoA, amino acids, and fatty acids metabolism were consistently found to be significantly enriched in the top enrichment analyses across Gene Ontology (GO), KEGG, WikiPathways and MP terms (Fig. 4*E*).

We subsequently got the protein-protein interaction network of these DEPs using STRING database and analyzed interaction degree by Cytoscape 3.9.1 (Supplemental Table S2). Interestingly, the top 50 DEPs with the highest interaction scores were mainly clustered in cell respiration, branched-chain amino acid (BCAA) degradation, and acyl-CoA metabolism (Fig. 3*F*). Together, these results suggest that HFD induces significant changes in the proteomic profile of VAT, reflecting characteristics of metabolic alteration, especially amino acid metabolism.

### HFD Induced Changes in the Ubiquitylome Profile of VAT

**Fig. 5:**
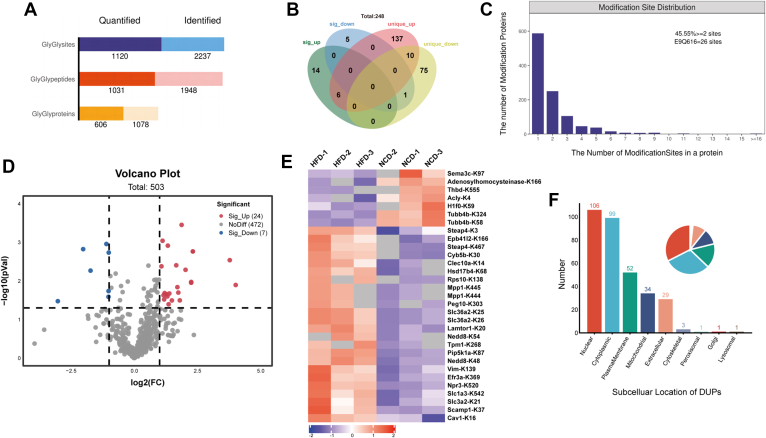
**Ubiquitylome profile changes of VAT from obese and lean mice.***A*, histogram displays the quantification and identification of ubiquitination modification sites, peptide and proteins in VAT of obese and lean mice. *B*, the Venn diagram illustrates the shared and unique DUPs in VAT of mice between HFD and NCD group. *C*, distribution of modification site across DUPs. The volcano plot (*D*) and heatmap (*E*) shows the differentially ubiquitylated sites, with statistically significant proteins indicated by triangular boxes [-log10 (*p*-value) ≥1.30, │fold change │≥2]. *F*, subcellular localization of the DUPs. VAT, visceral adipose tissue; DUPs, differentially ubiquitinated proteins; HFD, high-fat diet; NCD, normal chow diet. FC, fold change.

In the ubiquitylome analysis, a total of 1120 ubiquitylated lysine sites, 1031 ubiquitylated lysine peptides, 606 ubiquitylated lysine proteins were quantified (Fig. 5*A* and Supplemental Table S3). Among them, 153 proteins in HFD group and 86 proteins in NCD group underwent unique ubiquitination modification (Fig. 5*B*). 54.45% of the proteins exhibited a single ubiquitination site, while the EQ616 (AHNAK nucleoprotein) displayed the highest number of ubiquitination modification sites, with a total of 26 (Fig. 5*C*).

Using a cutoff criterion of a FC ≥2 and uncorrected *p* value < 0.05, we identified 24 up-regulated ubiquitinated Lys sites mapping to 20 proteins and seven down-regulated ubiquitinated Lys sites peptides mapping to six proteins (Fig. 5, *D* and *E*). These DUPs and unique ubiquitination modified proteins were majorly located in the cytoplasm (30.4%) and nucleus (32.5%), followed by the plasma membrane (15.9%) and mitochondrial (10.4%) (Fig. 5*F*).

**Fig. 6:**
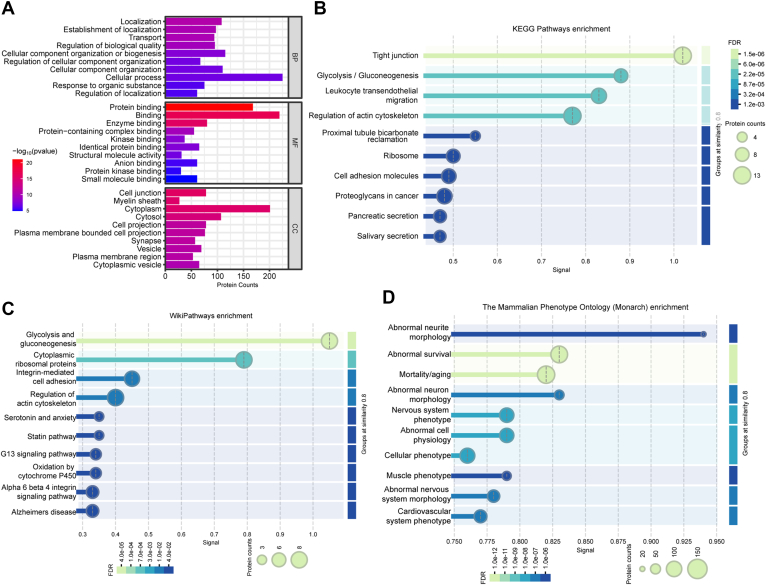
**Functional enrichment analysis of significantly differentially and uniquely ubiquitylated proteins in Ubiquitylome.** (*A*) gene ontology biological process enrichment analysis, (*B*) KEGG enrichment analysis, (*C*) WikiPathways and (*D*) Mammalian Phenotype Ontology enrichment analysis enrichment analysis of these DUPs and unique ubiquitination modified proteins. DUPs, differentially ubiquitinated proteins.

To further understand the implications of ubiquitination modifications in VAT during obesity, we conducted GO and KEGG enrichment analysis of these DUPs and unique ubiquitination modified proteins. The GO analysis showed that these proteins were significantly enriched in biological processes related to localization, transport and cellular component organization and so on (Fig. 6*A*). KEGG pathway analysis indicated significant enrichment of these proteins in pathways such as tight junction, glycolysis, gluconeogenesis, regulation of actin cytoskeleton, cell adhesion molecules and so on (Fig. 6*B*). WikiPathways enrichment analysis was related to regulation of actin cytoskeleton, glycolysis and gluconeogenesis, as well as the alpha six beta four integrin signaling pathway and G13 signaling pathway (Fig. 6*C*). Moreover, MP enrichment analysis suggested that these proteins were mainly related to abnormal survival, abnormal cell physiology, abnormal neurite morphology and so on (Fig. 6*D*).

### The Comparative Analysis of Proteome and Ubiquitylome of VAT Discovers Novel Potential Targets

**Fig. 7:**
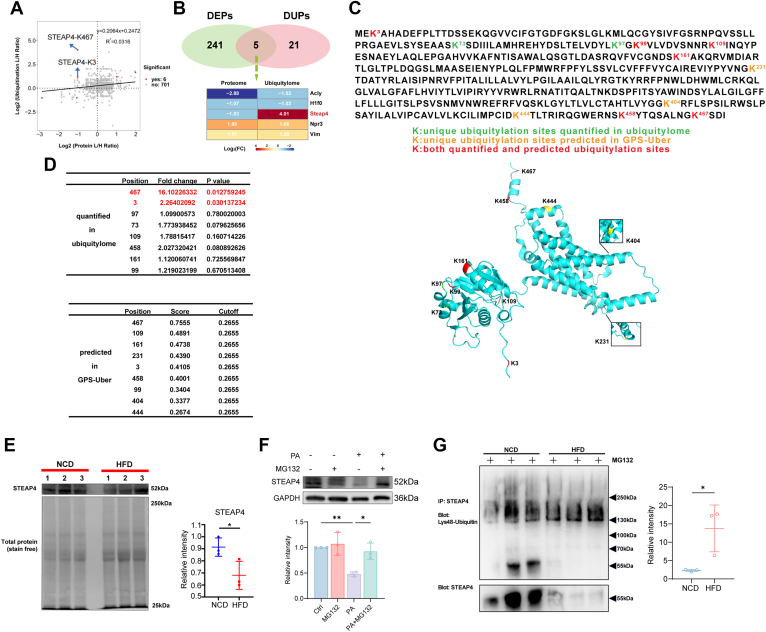
**HFD increases ubiquitination-mediated degradation of STEAP4 in VAT during obesity.***A*, scatterplot shows the correlation between the fold change of protein levels and ubiquitination modification levels. *B*, Venn diagram and heatmap depicts the overlapping protein among proteome and ubiquitylome. *C*, Amino acid sequences (*upper*) and 3D structure (*lower*) of STEAP4. *D*, Table of ubiquitination sites of STEAP4 quantified in our ubiquitylome (*upper*) and predicted by the GPS-Uber website (*lower*). *E*, Western blot analysis quantifies STEAP4 levels in VAT from mice fed HFD or NCD, normalized to total protein content using the stain-free method (n = 3 per group). *F*, Western blot analysis quantifies STEAP4 levels in 3T3-L1 adipocyte treated with PA (2 mM for 48 h) and MG132 (2 μM for 9 h before sample harvesting). The protein level was normalized to GAPDH (n = 3 per group). *G*, Validation of Lys48-linked ubiquitination modification levels of STEAP4 using Coimmunoprecipitation. Total protein was extracted from VAT of mice fed HFD or NCD in the presence of MG132 (10 μM). The VAT protein solution was immunoprecipitated with anti-STEAP4 antibodies. Eluted proteins were subjected to Western blot analysis and probed with specific antibodies against Lys48-specific ubiquitin and STEAP4 (n = 3 per group). The ubiquitination was normalized to pull down STEAP4. Data represent the mean ± SD. ∗*p* < 0.05 between indicated group; Unpaired *t* test was used for *E, F, G*. The results shown are representative of one of three independent experiments. DEPs, differently expressed proteins; DUPs, differentially ubiquitylated proteins; HFD, high-fat diet; NCD, normal chow diet; FC, fold change.

Then, we employed a scatter plot to visually illustrate the correlation between proteomic alterations and ubiquitination changes. The results revealed a weak positive correlation between protein expression levels and ubiquitination modification levels, suggesting that most variations in protein ubiquitination modification levels are likely attributed to changes in the expression of the proteins themselves (Fig. 7*A*). The Venn diagram revealed the overlapping DEPs and DUPs, and we identified five proteins that exhibited significant changes at both the protein level and the ubiquitination modification level. A subsequent heatmap displayed their fold-change patterns. Notably, STEAP4 was significantly downregulated at the protein level, while its ubiquitination was significantly upregulated (Fig. 7*B*). These results indicate that the downregulation of STEAP4 at the protein level is driven by enhanced ubiquitination. We then compared the ubiquitination sites of STEAP4 predicted by the GPS-Uber website (http://gpsuber.biocuckoo.cn) with the sites quantified in our ubiquitylome analysis (Fig. 7*D*) and displayed all the sites in a 3D structure map of STEAP4 (Fig. 7*C*). Among the quantified ubiquitination sites of STEAP4, only K3 and K467 exhibited significantly increased levels of ubiquitination in VAT from DIO mice, with K467 showing the largest increase in ubiquitination. Notably, this site also received the highest score according to the GPS-Uber prediction (Fig. 7*D*). This indicates that K467 is a critical site for the ubiquitination of STEAP4.

In order to verify the omics data, we performed *in vivo* and *in vitro* experiments, and found that VAT from DIO mice exhibited significantly reduced protein levels of STEAP4 compared to NCD-fed mice (Fig. 7*E*). Also, to mimic *in vivo* high-fat conditions, we treated 3T3-L1 adipocytes with palmitic acid (PA) for 48 h. The results showed that the PA treatment significantly reduced STEAP4 protein levels, whereas MG132 reversed this reduction (Fig. 7*F*). Further Co-immunoprecipitation (Co-IP) experiments confirmed that the level of STEAP4 protein in VAT of mice in the HFD group was significantly lower than that in the NCD group, but the K48-Ub signal intensities were comparable between the two groups, and the normalized statistical results showed that the K48-linked ubiquitination level of STEAP4 in VAT of mice in the HFD group was significantly increased (Fig. 7*G*). These results suggest that the upregulation of K48-linked ubiquitination of STEAP4 in VAT from DIO mice promotes proteasome-mediated protein degradation, leading to its downregulation at the protein level in obesity.

### Knockdown of STEAP4 in Adipocytes Reduces Mitochondrial Function

**Fig. 8:**
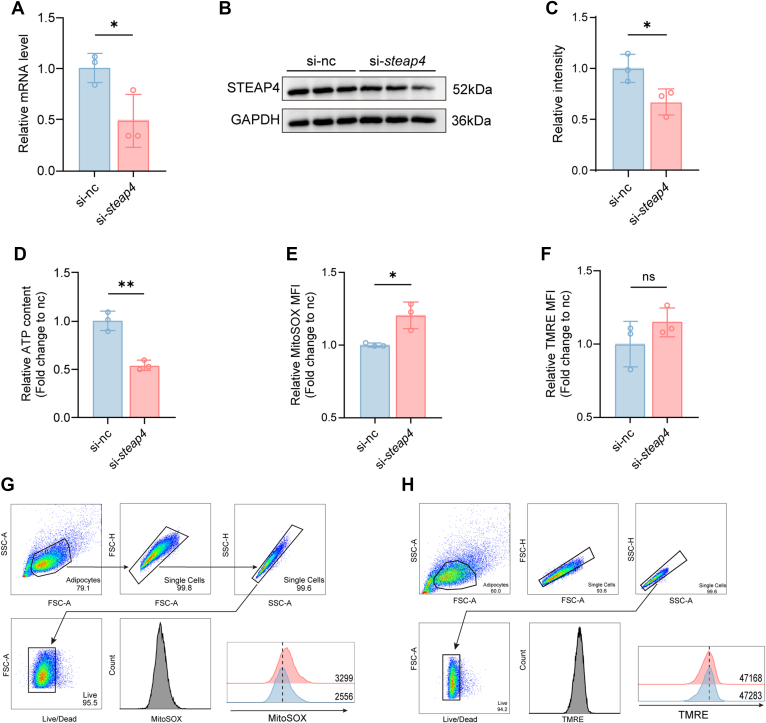
**Reduced SETAP4 expression in adipocytes impairs mitochondrial function.***A*, relative mRNA levels of *steap4* in 3T3-L1 adipocytes (*n* = 3) *B* and *C*, protein expressions and relative quantification in 3T3-L1 adipocytes (*n* = 3). *D*, ATP levels were expressed relative to total protein, determined by BCA assay after worm homogenization. *E* and *F*, MitoSOX signal (*E*) and TMRE (*F*) mean fluorescence intensity (MFI) assessed by flow cytometry. *G* and *H*, Representative flow cytometry plots for the analysis of MitoSOX signal MFI (*G*) and TMRE MFI (*H*) of adipocytes. Data represent the mean ± SD. ∗*p* < 0.05 and ∗∗*p* < 0.05 between indicated group; Unpaired *t* test was used for *A* and *C–F*. MFI, mean fluorescence intensity.

Recent studies have shown that STEAP4 orchestrates adipocyte mitochondrial function by sustaining electron-transport-chain activity, super-complex assembly and mitochondrial-gene splicing (13). However, impaired adipocyte mitochondrial function drives obesity and T2DM by suppressing lipid oxidation and thermogenesis, causing lipid accumulation and insulin resistance (14, 15). To validate the impact of STEAP4 downregulation on mitochondrial function in adipocytes, we knocked down the STEAP4 expression at both the gene and protein levels in 3T3-L1 adipocyte *in vitro* using siRNA (Fig. 8, *A–C*). The results demonstrated that STEAP4 knockdown led to a decrease in ATP content in 3T3-L1 adipocytes and an elevation in mitochondrial ROS levels, with no significant effect on mitochondrial membrane potential (Fig. 8, *D–F*). The representative flow plots and the gating strategy are shown in Figure 8, *G* and *H*. These findings confirm that reduced STEAP4 expression impairs mitochondrial function of adipocytes. Therefore, we hypothesize that during obesity, STEAP4 in adipocytes undergoes accelerated degradation due to enhanced ubiquitination modification, leading to a decrease in its protein level. This, in turn, impairs mitochondrial function in adipocytes, ultimately exacerbating adipocyte hypertrophy and metabolic disorders.

## Discussion

Ubiquitination has received increasing attention due to its pivotal role in protein degradation and the regulation of various cellular events. Recent studies have suggested that ubiquitination has a potential regulatory role in the obesity-related inflammation or metabolic disorders (12, 16, 17). However, the integrated analysis of the proteome and ubiquitylome of VAT in obesity were still unexplored. In this study, we conducted an integrated comparative analysis of proteome and ubiquitylome of lean or obese VAT, and revealed that STEAP4 in VAT may be involved in HFD-induced obesity through abnormal ubiquitination modification.

In a previous study, utilizing LC-MS/MS and extended multiplexing with tandem mass tags labeling, Plubell DL *et al.* had analyzed proteomes of epiWAT of mice fed either HFD or NCD for eight or 18 weeks (7).They identified 43 differential proteins of 8-weeks HFD fed mice and found that a short-term HFD resulted in significant disruptions in lipid, carbohydrate, BCAA and mitochondrial metabolic pathways. Here, we identified 357 differential proteins and also revealed a significant change of BCAA, and lipid metabolism. It’s clear that abnormalities in lipid metabolism are related to IR (18, 19). Moreover, the circulating levels of BCAAs are positively correlated with IR in T2DM patients and obese mice (20). In contrast, during weight loss, plasma BCAA levels decrease in tandem with improvements in insulin sensitivity (21). Interestingly, obese mice showed the decreased levels of BCAAs in WAT (22), possibly due to reduced BCAA utilization requirements in WAT in obesity. These observations collectively suggest that disruptions in BCAA metabolism may be crucially involved in the development of obesity and IR. Here, we also found that acetyl-CoA metabolic process was significantly altered in VAT from DIO mice. Acetyl-CoA serves as a central hub in cellular metabolism, participating in multiple pathways such as glycolysis, fatty acid synthesis and oxidation, and ketogenesis (23). Dysregulation of acetyl-CoA levels can lead to metabolic disorders. Moreover, Acetyl-CoA plays a crucial role in regulating gene expression through histone acetylation, a post-translational modification that affects chromatin structure and transcriptional activity (23). This mechanism can influence obesity-related metabolic pathways, such as lipid metabolism and glucose homeostasis, thereby contributing to the development of obesity and associated metabolic disorders.

By comparing the quantitative results of the ubiquitylome and proteome, we found that STEAP4 exhibited decreased protein levels but increased ubiquitination modification levels in VAT under HFD conditions, suggesting that changes in these protein levels may be related to abnormal ubiquitination mediated protein degradation in VAT during obesity. However, our analysis of the proteome and ubiquitylome in VAT was designed to identify proteins potentially regulated by ubiquitination during obesity. Instead of normalizing ubiquitination levels to total protein abundance, we focused on detecting the proteins exhibiting opposite change trend between protein levels and ubiquitination levels, as this pattern is highly indicative of proteasome-dependent ubiquitin-mediated protein regulation. This approach is particularly suitable for initial screening of functional ubiquitination targets from large-scale datasets, as it highlights candidates where ubiquitination may actively regulate protein stability. A notable example is STEAP4, whose significant downregulation at the protein level alongside marked upregulation in ubiquitination strongly supports PTMs regulation *via* the ubiquitin-proteasome system. However, we acknowledge that without protein-level normalization, our ability to interpret changes in proteins with concordant trends remains limited. Future studies incorporating normalized ubiquitylomic quantifications will help further distinguish whether changes in ubiquitination sites are driven primarily by alterations in protein abundance or reflect genuine post-translational regulation.

We also observed a significant downregulation of STEAP4 in the proteome of the HFD group compared with the NCD group; however, the FC in the IP assay appeared much higher than that in the proteome. We propose that there may be three underlying reasons: first, this divergence likely stems from a fundamental methodological difference in detection scope. Mass spectrometry-based proteomics quantifies the total pool of STEAP4 peptides, providing an unbiased measure of protein levels (24), in contrast, IP efficiency is inherently dependent on antibody-epitope interaction, selectively enriching only the subset of STEAP4 molecules whose specific epitopes are accessible for antibody binding. Second, STEAP4 undergoes significantly increased ubiquitination in the VAT of DIO mice. These bulky ubiquitin chains impose steric hindrance, impairing antibody-epitope binding and diminishing affinity (25), this would consequently impair IP enrichment in the HFD group, resulting in weaker Western blot bands. Third, it is also plausible that batch-to-batch variations in samples processed for proteomic analysis *versus* IP could underlie this quantitative divergence; notably, however, the directional trend of STEAP4 abundance remains completely consistent across both approaches, confirming the reliability of the core observation.

STEAP4 (six transmembrane epithelial antigen of the prostate 4), as a metal reductase, can reduce ferric iron (Fe^3+^) to ferrous iron (Fe^2+^) to promote cellular iron absorption. Meanwhile, it is also known as tumor necrosis factor α-induced adipose-related protein, and its expression is regulated by inflammatory factors such as growth hormone, interleukin-6 (IL-6), and tumor necrosis factor-α (TNF-α) (26, 27, 28). This molecule plays a key role in the regulation of obesity and insulin resistance. STEAP4 can inhibit adipogenesis through a PPAR-γ-dependent mechanism, thereby preventing obesity and metabolic disorders (29). And another study showed that STEAP4 knockdown in adipocytes may impair mitochondrial function, thereby promoting obesity-related metabolism dysfunction (13). Clinical studies confirmed that the expression level of STEAP4 protein is negatively correlated with BMI, and the level of STEAP4 protein in adipose tissue of obese individuals is significantly downregulated (28, 29). Existing studies have clearly shown that STEAP4 deficiency leads to adipose tissue dysfunction, enhanced inflammation, and impaired insulin signaling (30, 31, 32), while overexpression can improve insulin resistance (33).

However, although existing studies have clarified the protective effect of STEAP4 on metabolic health and the characteristic that STEAP4 protein level in adipose tissue is significantly downregulated under obese conditions, the mechanism underlying the decrease in its protein level remains unclear. Through the analysis of proteomics and ubiquitomics data, combined with co-IP and *in vitro* verification, this study reveals that the key mechanism for the downregulation of STEAP4 protein expression in obese adipose tissue is related to the significant increase in its Lys48 ubiquitination modification level, which in turn promotes proteasome-dependent degradation.

To verify the core mechanism by which the downregulation of STEAP4 expression in adipocytes promotes obesity, we interfered with STEAP4 expression in 3T3-L1 adipocytes through siRNA, and confirmed that its downregulation can directly lead to mitochondrial dysfunction, manifested as increased mitochondrial ROS production and decreased ATP production. Given that adipocyte mitochondrial dysfunction is generally regarded as a core link in obesity-related metabolic disorders, it can damage insulin signaling through ROS accumulation, induce chronic inflammation, and thereby promote the progression of obesity and related complications (14, 15, 34, 35). Therefore, our data support that the downregulation of STEAP4 promotes obesity and related metabolic disorders by impairing adipocyte mitochondrial function, which is highly consistent with previous reports.

In conclusion, this study not only clarifies that the STEAP4 protein level under obese conditions is regulated by the ubiquitin-proteasome pathway but also suggests that the downregulation of STEAP4 may promote obesity-related metabolic disorders by inducing adipocyte mitochondrial dysfunction, providing a new molecular perspective and theoretical basis for the pathogenesis of obesity-related metabolic diseases. However, the upstream mechanism by which obesity promotes STEAP4 ubiquitination modification in adipocytes requires further research.

## Limitations

• The regulatory mechanism underlying the increased ubiquitination of STEAP4 in VAT during obesity remains to be elucidated.
• Model scope: The study was conducted exclusively in male mice, requiring validation in human samples to generalize conclusions.
• Technical and analytical constraints: Potential detection depth limitations exist due to the specific ubiquitin enrichment kit used.

## Data Availability

All data from this study are available upon. The mass spectrometry proteomics and ubiquitylomics data have been deposited to the ProteomeXchange Consortium *via* the PRIDE (36) partner repository with the dataset identifier PXD070339.

## Supplemental data

This article contains supplemental data.

## Conflict of interest

The authors declare no competing interests.
